# Association Between Physical Activity and Cryptogenic Ischemic Stroke in Young Adults: A Case–Control Study

**DOI:** 10.1111/ene.70650

**Published:** 2026-05-28

**Authors:** Shakar Kutal, Nilufer Yesilot, Tomi Sarkanen, Petra Redfors, Annika Nordanstig, Pauli Ylikotila, Marialuisa Zedde, Ulla Junttola, Lauri Tulkki, Kristina Ryliskiene, Radim Licenik, Phillip Ferdinand, Dalius Jatužis, Liisa Kõrv, Janika Kõrv, Alessandro Pezzini, Juha Sinisalo, Mika Lehto, Eva Gerdts, Jaana Autere, Ana Catarina Fonseca, André Paula, Ulrike Waje‐Andreassen, Bettina von Sarnowski, Tiina Sairanen, Turgut Tatlisumak, Juha Huhtakangas, Pekka Jäkälä, Jukka Putaala, Nicolas Martinez‐Majander

**Affiliations:** ^1^ Department of Neurology Helsinki University Hospital, and University of Helsinki Helsinki Finland; ^2^ Department of Neurology Istanbul University Faculty of Medicine Istanbul Turkey; ^3^ Department of Neurology, Tampere University Hospital, Wellbeing Services County of Pirkanmaa, and Faculty of Medicine and Health Technology Tampere University Tampere Finland; ^4^ Department of Neurology, Sahlgrenska University Hospital and Department of Clinical Neuroscience, Institute of Neuroscience and Physiology Sahlgrenska Academy at University of Gothenburg Gothenburg Sweden; ^5^ Department of Neurology, Neurocenter University of Turku Turku Finland; ^6^ Neurology Unit Azienda Unità Sanitaria Locale‐IRCCS Di Reggio Emilia Reggio Emilia Italy; ^7^ Department of Neurology, Faculty of Medicine, Neurocenter Oulu University Hospital, Finland and Research Unit of Clinical Medicine Oulu Finland; ^8^ Centre of Neurology, Faculty of Medicine Vilnius University Vilnius Lithuania; ^9^ North West Anglia NHS Foundation Trust Acute Stroke Centre Peterborough UK; ^10^ Neurosciences University Hospitals of North Midlands NHS Trust Stoke‐on‐Trent UK; ^11^ Department of Neurology and Neurosurgery University of Tartu Tartu Estonia; ^12^ Department of Medicine and Surgery, Department of Emergency Parma University Hospital, University of Parma and Stroke Care Program Parma Italy; ^13^ Department of Cardiology Helsinki University Hospital and University of Helsinki Helsinki Finland; ^14^ Department of Internal Medicine, Jorvi Hospital HUS Helsinki University Hospital, and University of Helsinki Helsinki Finland; ^15^ Department of Heart Disease Haukeland University Hospital Bergen Norway; ^16^ Department of Clinical Science University of Bergen Bergen Norway; ^17^ Neurocenter Neurology Kuopio University Hospital, Finland and University of Eastern Kuopio Finland; ^18^ Department of Neurosciences and Mental Health (Neurology), hospital de Santa Maria‐CHLN, Faculdade de Medicina Universidade de Lisboa Lisboa Portugal; ^19^ Department of Neurology Haukeland University Hospital Bergen Norway; ^20^ Department of Neurology University Medicine Greifswald Greifswald Germany

**Keywords:** ischemic stroke, patent foramen ovale, physical activity, risk factors, stroke in the young

## Abstract

**Background:**

Data on the association between physical activity (PA) and cryptogenic ischaemic stroke (CIS) in young adults are scarce. We investigated the relationship between PA levels and early‐onset CIS, stratified by the presence of a high‐risk patent foramen ovale (PFO).

**Methods:**

Patients aged 18–49 years with first‐ever CIS and sex‐ and age‐matched stroke‐free controls were recruited from 19 European centres. PA was assessed using the short International Physical Activity Questionnaire and expressed as Metabolic Equivalents, categorized into percentiles (bottom 10%, 10%–25%, 25%–75% [reference], 75%–90%, top 10%). Associations between PA and CIS were analyzed using conditional logistic regression adjusted for age, education level, traditional risk factors, and non‐traditional risk factors.

**Results:**

Altogether, 533 patients (median age 41 [interquartile range 34–46]; 47.3% women) and 533 controls were included. Scoring in the top 10% of PA was independently associated with CIS: adjusted odds ratio 2.07; 95% confidence interval 1.22‐3.51. Comparing patients without high‐risk PFO to all controls, the top 10% PA category (1.78; 1.07‐2.94) and the bottom 10% category (1.76; 1.06‐2.92) were associated with CIS. No independent association was observed in patients with high‐risk PFO.

**Conclusions:**

PA in the top 10% was associated with an increased risk of early‐onset CIS in the overall study population. Among patients without a high‐risk PFO, both the bottom 10% and top 10% of PA were consistently associated with elevated CIS risk. These findings highlight the need to better understand the mechanisms through which both low and high activity levels may predispose to stroke.

**Nonstandard Abbreviations and Acronyms**
CIS: Cryptogenic ischemic stroke
MET: Metabolic equivalent
PA: Physical activity
PFO: Patent foramen ovale
SECRETO: Searching for Explanations for Cryptogenic Stroke in the Young: Revealing the Triggers, Causes, and Outcome

## 1 Introduction

In recent years, several large‐scale studies have confirmed a high burden of traditional vascular risk factors in young adults who experience ischemic stroke (IS) [1, 2, 3]. On the other hand, a prospective population‐based study observed that the rising incidence of IS among young adults is primarily driven by cases without clear vascular explanations, pointing to the relevance of emerging and underexplored factors in stroke pathogenesis at younger ages [4].

Low physical activity (PA) and hypertension have been identified as the strongest contributors to early‐onset IS, with smoking and binge drinking as well as heavy alcohol consumption also contributing substantially [5, 6]. In addition, migraine with aura (MA) has been associated with cryptogenic ischemic stroke (CIS) in young adults, further underlining the need to examine non‐traditional contributors in this population [7]. Importantly, up to 40% of IS in the young are classified as cryptogenic, making CIS the most common etiologic subtype in this age group [8]. More recently, self‐perceived psychological stress has emerged as a potential risk factor for early‐onset IS, with studies reporting elevated risk estimates even after accounting for vascular comorbidities [9, 10]. These findings suggest that behavioral and lifestyle‐related exposures may play a disproportionately large role in younger individuals compared to older stroke patients [11].

PA may represent another key factor in explaining IS among younger individuals, given evolving lifestyle patterns characterized by both physical inactivity and, in some cases, excessive training. However, defining PA using a single cut‐off value is problematic, as such thresholds often fail to capture the complexity of different activity forms (intensity, frequency, and type), and may vary considerably with age [12]. Sedentary behavior has become increasingly common due to occupational sitting, reduced active commuting, and leisure‐time screen exposure, potentially contributing to strokes of all types [13]. In addition to low PA, extreme PA has been associated with a transiently increased risk of IS in young adults within the hour following exertion [14]. In individuals with a patent foramen ovale (PFO), peak exercise may precipitate a right‐to‐left shunt due to fluctuations in right atrial and pulmonary arterial pressures [15]. In fact, one study found a significant association between vigorous exercise and PFO‐related stroke in young adults, reporting an odds ratio of 3.4 [16].

It is necessary to uncover the contributing factors of early‐onset CIS as conventional risk factors do not fully account for the risk in this age group. In this prospective international case–control study, we aimed to evaluate the relationship between different levels of PA, including low and vigorous, as well as sedentary behavior, and the risk of early‐onset CIS.

## 2 Methods

### 2.1 Study Population

Between November 2013 and November 2022, a total of 546 young patients with CIS and 546 stroke‐free controls, matched for age and sex, were enrolled from 19 European centers participating in the prospective multicenter Searching for Explanations for Cryptogenic Stroke in the Young: Revealing the Etiology, Triggers, and Outcome (SECRETO; NCT01934725) study. Eligible patients were aged 18 to 49 years and had been hospitalized for a first‐ever CIS confirmed by imaging [17]. Inclusion criteria required evidence of an acute ischemic lesion on diffusion‐weighted magnetic resonance imaging or arterial occlusion and perfusion deficit corresponding with acute symptoms. Silent brain infarcts and prior transient ischemic attacks were permitted. Patients were excluded if essential baseline investigations, including brain magnetic resonance imaging and standard laboratory tests, were not completed within the first week post‐stroke, or if additional diagnostic evaluations (such as imaging of cervicocephalic arteries, echocardiography, electrocardiogram, 24 h Holter monitoring, or thrombophilia testing) were not performed within the first 2 weeks. This study follows the STROBE (Strengthening the Reporting of Observational Studies in Epidemiology) reporting guideline (www.strobe‐statement.org).

All enrolled patients received a thorough and standardized diagnostic evaluation to exclude known causes of stroke. This assessment included brain magnetic resonance imaging, imaging of extracranial and intracranial arteries via computed tomography angiography or magnetic resonance angiography, and laboratory testing in accordance with the study protocol. Furthermore, all participants underwent 12‐lead electrocardiogram and continuous electrocardiogram monitoring for a minimum of 24 h, as well as both transthoracic and transesophageal echocardiography performed according to a standardized approach [18]. Patients with individual coagulopathies were not excluded, given the uncertain causal relationship, except in cases of previously diagnosed antiphospholipid antibody syndrome. Additional investigations, including transcranial Doppler with bubble contrast, were performed at selected sites. Stroke severity was assessed using the National Institutes of Health Stroke Scale (NIHSS) score.

CIS was classified according to the A‐S‐C‐O system, defined as either the absence of a detectable cause (grade 0) or the presence of a condition with uncertain causality (grade II) or a condition considered unlikely to be the direct cause (grade III), as determined by the most reliable diagnostic evidence available [19].

For each patient, a stroke‐free control matched by sex and age (±5 years) from the same geographical area was recruited at the corresponding study center. Control participants were identified using several approaches, such as random selection from population registries, when possible, recruitment of unrelated acquaintances of patients, and inclusion of hospital staff not involved in the study. Owing to differences in legal and procedural requirements across sites, recruitment strategies for controls were not entirely uniform. Eligibility required no prior history of stroke, verified using the Questionnaire for Verifying Stroke‐Free Status [20] and by review of medical records. Whenever feasible, controls were enrolled within 3 months of the corresponding patient to reduce the risk of temporal confounding.

### 2.2 Standard Protocol Approvals, Registrations, and Patient Consents

The study protocol was approved by the Ethics Committee of the Helsinki University Hospital (HUS/2684/2017), which acted as the lead ethical review board. Furthermore, the study was approved by the institutional review boards or ethics committees of all other participating centers in accordance with local regulations. Written informed consent was obtained from all participants prior to enrollment. Data supporting the findings are available upon reasonable request.

### 2.3 Cardiovascular Risk Factors and Comorbidities

The studied vascular and lifestyle risk factors were defined according to standard clinical criteria (see Supporting Information and Table S1 for detailed definitions and measurement procedures).

PA was assessed using the short form of the International Physical Activity Questionnaire [21], which records participants' weekly energy expenditure in metabolic equivalents (METs). The questionnaire comprises six items capturing the frequency and duration of PA at three intensity levels—vigorous, moderate, and walking—during a typical week over the past year. The total MET‐minutes per week were calculated and categorized into percentiles based on the values obtained from controls: bottom 10% (METs 0–693), 10%–25% (METs 694–1539), 25%–75% (METs 1540–4746), 75%–90% (METs 4747–8062), and top 10% (METs > 8062), with the middle 25%–75% percentile group serving as the reference category representing at least moderate but not extremely vigorous PA. The same questionnaire asks participants to report their average daily time spent sitting (recorded in hours). This item had no effect on the calculated MET. Time spent sitting was analyzed as a continuous variable descriptively and dichotomized in regression models using a ≥ 10 h cut‐off (sedentary behavior), consistent with prior literature [22].

In this study, a clinically significant high‐risk PFO was defined as the presence of a PFO accompanied by an atrial septal aneurysm detected on transesophageal echocardiography, or the identification of a large right‐to‐left shunt on either transesophageal echocardiography or transcranial Doppler bubble study [18, 23].

### 2.4 Statistical Analysis

Statistical analyses were performed using conditional logistic regression appropriate for the matched case–control design. Missing data for selected variables were imputed using multivariable imputation by chained equations (details in Supporting Information). Baseline characteristics of cases and controls were compared using McNemar's test for categorical and paired t‐ or Wilcoxon signed‐rank tests for continuous variables. Associations between PA levels and CIS were examined in three models adjusted for (1) age, sex, and education; (2) traditional vascular risk factors; and (3) additional non‐traditional risk factors. Traditional vascular risk factors included hypertension, current smoking, abdominal obesity, heavy alcohol use, depression, unhealthy diet, and stress. Non‐traditional vascular risk factors included migraine with aura, chronic multi‐organ disorder, and illicit drug use. Diabetes, hypercholesterolemia, cardiovascular disease, obstructive sleep apnea, venous thromboembolism, and malignancy were not included in the regression models due to their low prevalence in the study population.

Subgroup analyses stratified by sex, age, and high‐risk PFO status, and a sensitivity analysis using exclusively population‐based controls, were also conducted. Analyses were performed in International Business Machines Statistical Package for the Social Sciences Statistics (version 29.0.2) and RStudio (version 2024.04.2 + 764), and two‐tailed *p*‐values < 0.05 were considered statistically significant.

## 3 Results

Of the initial 546 matched case–control pairs, we included 533 patients (median age 41 years, IQR 34–46; 47.3% women) and 533 age‐ and sex‐matched controls with PA data available. Among patients, the median delay from hospital admission to study inclusion was 6 days (IQR 3–10). Median NIHSS score on admission was 2 (IQR 0–4, range 0–35). Of all patients, 25.8% had NIHSS score 0, 50.4% had mild strokes (NIHSS 1–4), 14.0% moderate strokes (NIHSS 5–9), and 9.8% severe strokes (NIHSS ≥ 10). Stroke severity did not differ between patients with very low and extremely high levels of PA (*p* = 0.850). Baseline characteristics across the PA categories for all patients are presented in Table S2. Differences in risk factors between female and male patients, and patients aged 18–39 years and 40–49 years, are shown in Table S3.

### 3.1 Univariable Comparison Between Patients and Matched Controls

**FIGURE 1:**
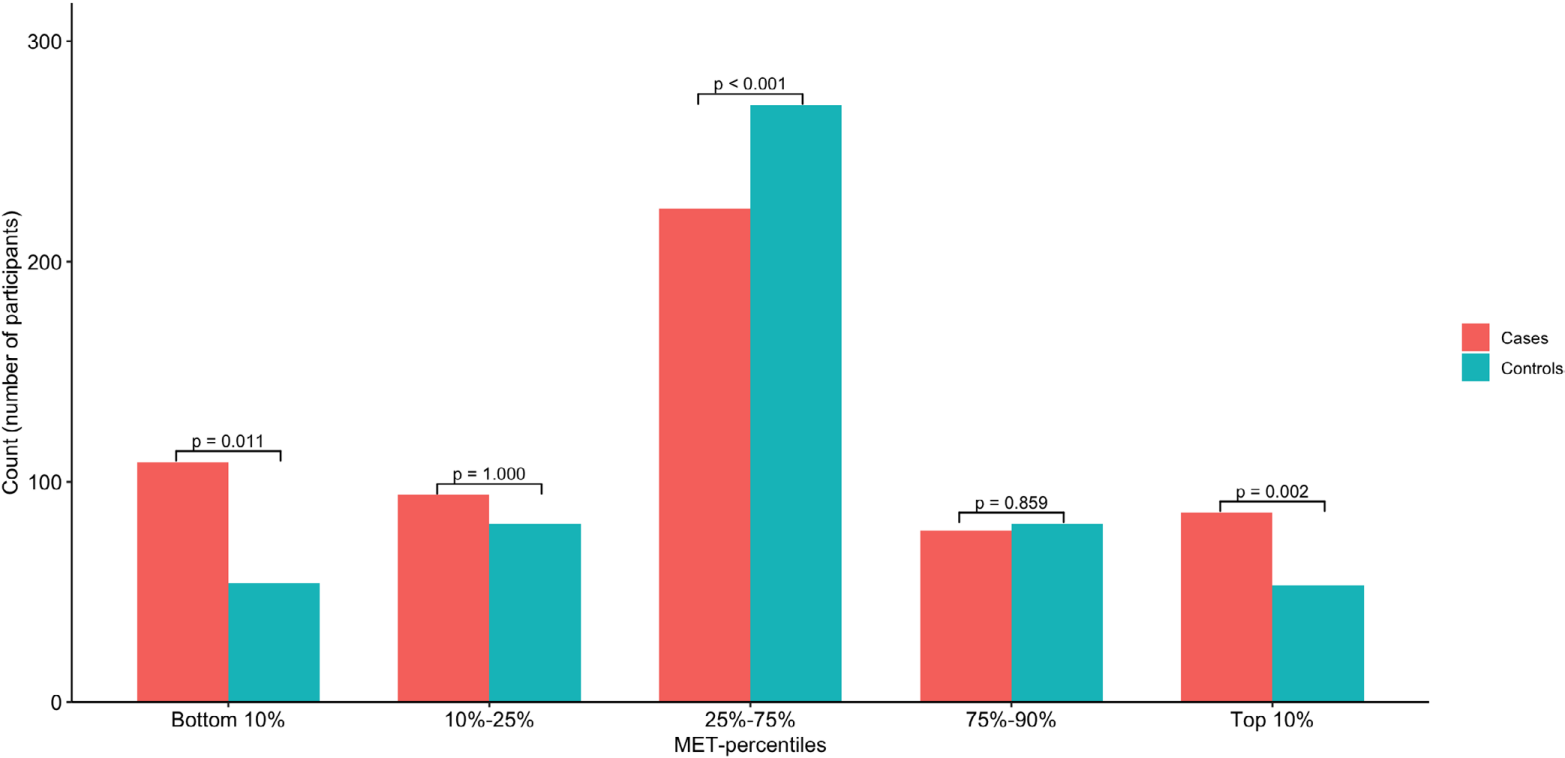
Distribution of Metabolic Equivalent categories for cases and controls.

Apart from PA and sedentary behavior, the clinical characteristics of CIS patients and stroke‐free controls are presented in Table 1. Compared with controls, patients were less educated and had more often hypertension, cardiovascular disease, current smoking, abdominal obesity, heavy alcohol consumption, depression, unhealthy diet, stress related to home or work, MA, and history of venous thromboembolism. With respect to PA, patients in the overall study population were more likely to be either physically inactive or, conversely, engage in very high levels of activity compared to controls (Figure 1).

**TABLE 1:** Baseline characteristics of young cryptogenic ischemic stroke cases and stroke‐free control subjects included in the study.

Characteristic (number of cases/controls with missing data)	All			Women			Men		
	Cases (*n* = 533)	Controls (*n* = 533)	*p*‐value	Cases (*n* = 252)	Controls (*n* = 252)	*p*‐value	Cases (*n* = 281)	Controls (*n* = 281)	*p*‐value
**Age**	41 (34–46)	42 (34–46)	N/A	40 (31–45)	41 (31–45)	N/A	42 (36–46)	42 (36–47)	N/A
Low level of education (2/0)	294 (55.4)	184 (34.5)	< 0.001	131 [52.2]	84 [33.3]	< 0.001	163 [58.2]	100 [35.6]	< 0.001
Hypertension (0/4)	184 [34.5]	142 [26.8]	0.007	80 [31.7]	53 [21.3]	0.010	104 [37.0]	89 [31.8]	0.210
Diabetes mellitus (0/1)	16 [3.0]	10 [1.9]	0.327	6 [2.4]	0 [0.0]	N/A	10 [3.6]	10 [3.6]	1.000
Cardiovascular disease^†^	15 [2.8]	5 [0.9]	0.041	5 [2.0]	2 [0.8]	0.453	10 [3.6]	3 [1.1]	0.092
Current tobacco smoking (2/1)	173 [32.6]	77 [14.5]	< 0.001	68 [27.1]	37 [14.7]	< 0.001	105 [37.5]	40 [14.3]	< 0.001
Hypercholesterolemia	12 [2.3]	24 [4.5]	0.043	2 [0.8]	4 [1.6]	0.687	10 [3.6]	20 [7.1]	0.064
Abdominal obesity^‡^ (0/6)	315 [59.1]	235 [44.6]	< 0.001	108 [42.9]	63 [25.3]	< 0.001	207 [73.7]	172 [61.9]	0.001
Heavy alcohol use (0/1)	70 [13.1]	31 [5.8]	< 0.001	29 [11.5]	18 [7.1]	0.090	41 [14.6]	13 [4.6]	< 0.001
Depression (1/5)	163 [30.6]	121 [22.9]	0.006	84 [33.3]	71 [28.4]	0.259	79 [28.1]	50 [18.0]	0.006
Unhealthy diet (2/1)	272 [51.2]	193 [36.3]	< 0.001	117 [46.6]	72 [28.7]	< 0.001	155 [55.4]	121 [43.1]	0.004
Obstructive sleep apnea (4/7)	13 [2.5]	12 [2.3]	1.000	1 [0.4]	3 [1.2]	0.625	12 [4.3]	9 [3.3]	0.824
Work or home related stress (0/1)	265 [49.7]	217 [40.8]	0.003	132 [52.4]	117 [46.6]	0.195	133 [47.3]	100 [35.6]	0.005
Migraine with aura (0/1)	220 [41.3]	92 [17.3]	< 0.001	127 [50.4]	57 [22.7]	< 0.001	93 [33.1]	35 [12.5]	< 0.001
Chronic multiorgan disorder^§^ (1/1)	76 [14.3]	55 [10.3]	0.050	47 [18.7]	33 [13.1]	0.103	29 [10.3]	22 [7.8]	0.349
Venous thromboembolism (2/0)	18 [3.4]	4 [0.8]	0.004	10 [4.0]	2 [0.8]	0.039	8 [2.9]	2 [0.7]	0.109
Malignancy	10 [1.9]	8 [1.5]	0.815	4 [1.6]	6 [2.4]	0.754	6 [2.1]	2 [0.7]	0.289
Illicit drug use (0/1)	41 [7.7]	27 [5.1]	0.092	16 [6.3]	10 [4.0]	0.307	25 [8.9]	17 [6.0]	0.243

*Note:* Data are *n* [%] or median (interquartile range). N/A, not applicable.

^†^ Cardiovascular disease includes any of the following: coronary heart disease, chronic heart failure, peripheral artery disease, history of myocardial infarction, arterial thrombosis, aneurysm, or aortic or valvular diseases.

^‡^ Waist‐to‐hip ratio > 0.85 in women, > 0.90 in men.

^§^ Chronic multiorgan disorder includes any of inflammatory bowel disease (IBD), autoimmune disease (excluding IBD), chronic kidney or liver disease, or hematologic disease or thrombophilia.

Among women, patients were more often current smokers, had lower education, unhealthy diet, obesity, hypertension, MA, and history of venous thromboembolism. Male patients showed similar differences and were additionally more likely to report heavy alcohol use, depression, and stress related to work or home (Table 1).

Regarding sex‐specific results, female patients were more frequently physically inactive compared to female controls. In contrast, male patients were more often classified as extremely active than their control counterparts (Table S4). When analyzed by age, no significant differences in PA were observed among patients younger than 40 years. However, in the 40–49 year‐olds, patients were more likely to be either physically inactive or extremely active compared to controls (Table S5).

**FIGURE 2:**
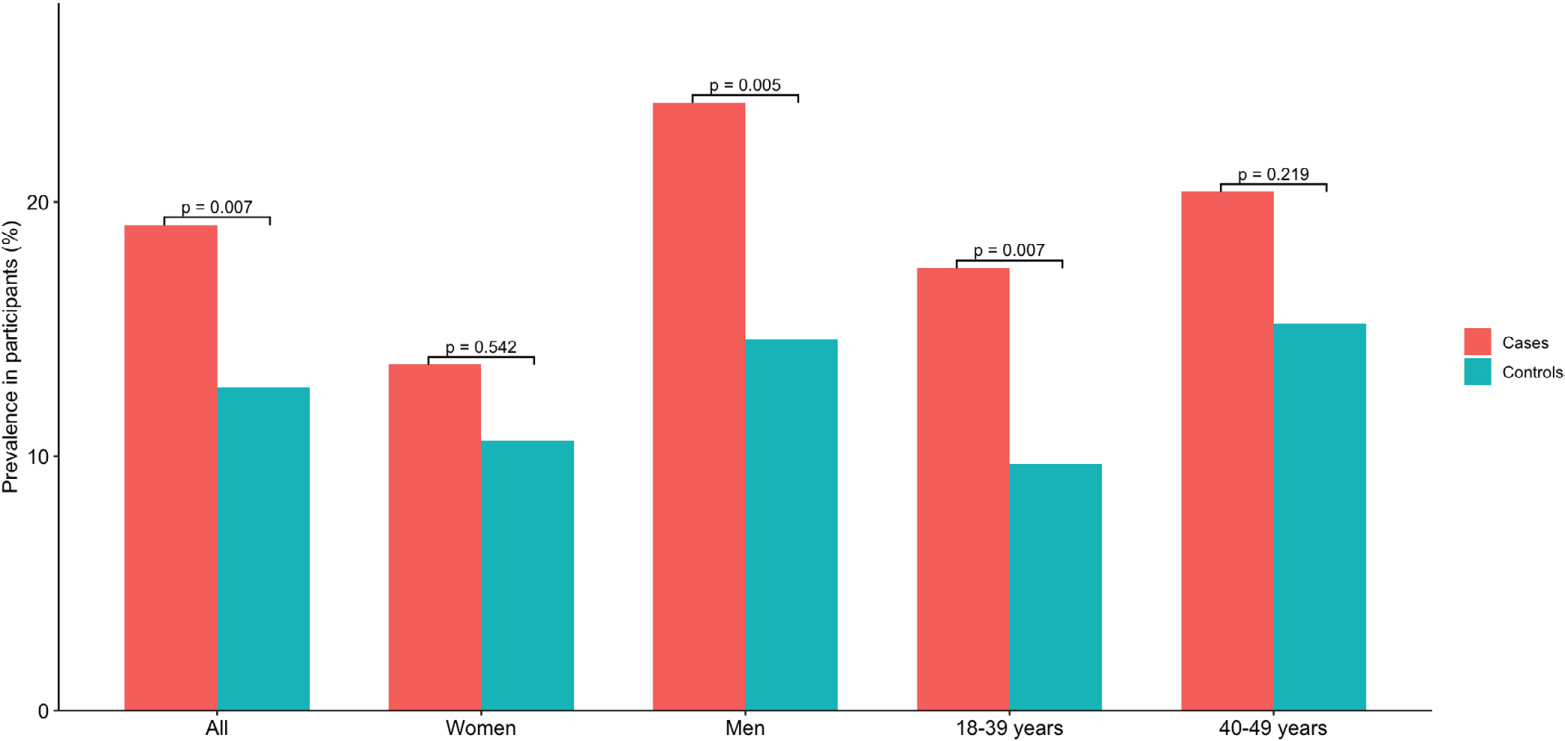
Prevalence in participants with ≥ 10 h of daily sitting, stratified by sex and age group.

In terms of sedentary behavior, patients were more likely to report sitting for 10 h or more per day compared to controls. This difference was also evident when analyses were stratified by sex, with male patients showing higher prevalence than male controls, and by age, with patients younger than 40 years more likely to exceed the 10 h threshold than their age‐matched counterparts (Figure 2).

### 3.2 Association Between PA and CIS

**FIGURE 3:**
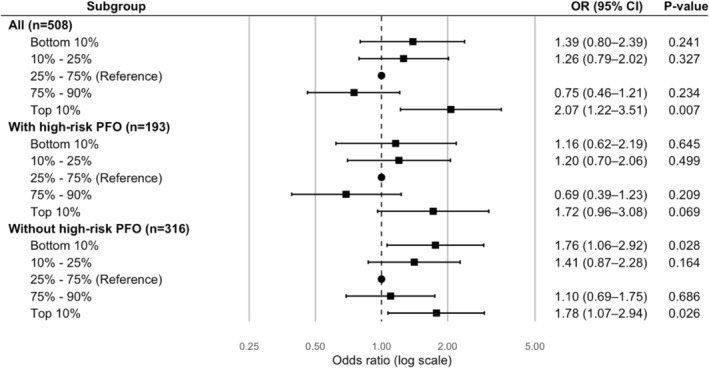
Association between physical activity and cryptogenic ischemic stroke, stratified by patent foramen ovale (PFO) status. OR, odds ratio; CI, confidence interval. Model 3, fully adjusted for demographics, traditional and non‐traditional vascular risk factors.

After adjusting for age, sex, and education, PA showed a significant association with early‐onset CIS in the overall study population, specifically among individuals in both the bottom 10% and top 10% of METs. Following additional adjustment for traditional and non‐traditional risk factors, the association in the whole cohort persisted only for those in the top 10% of METs (Table S6, Figure 3).

In high‐risk PFO cases compared to all controls, a significant association was found between early‐onset CIS and being in the top 10% of METs when adjusted for demographics and traditional risk factors. However, this association did not remain significant in the fully adjusted model (Table S6, Figure 3).

Among cases without a high‐risk PFO, significant associations with early‐onset CIS were observed for both the bottom 10% and top 10% of METs. These associations remained significant even in the fully adjusted model (Table S6, Figure 3).

### 3.3 Association Between PA and CIS According to Sex

**FIGURE 4:**
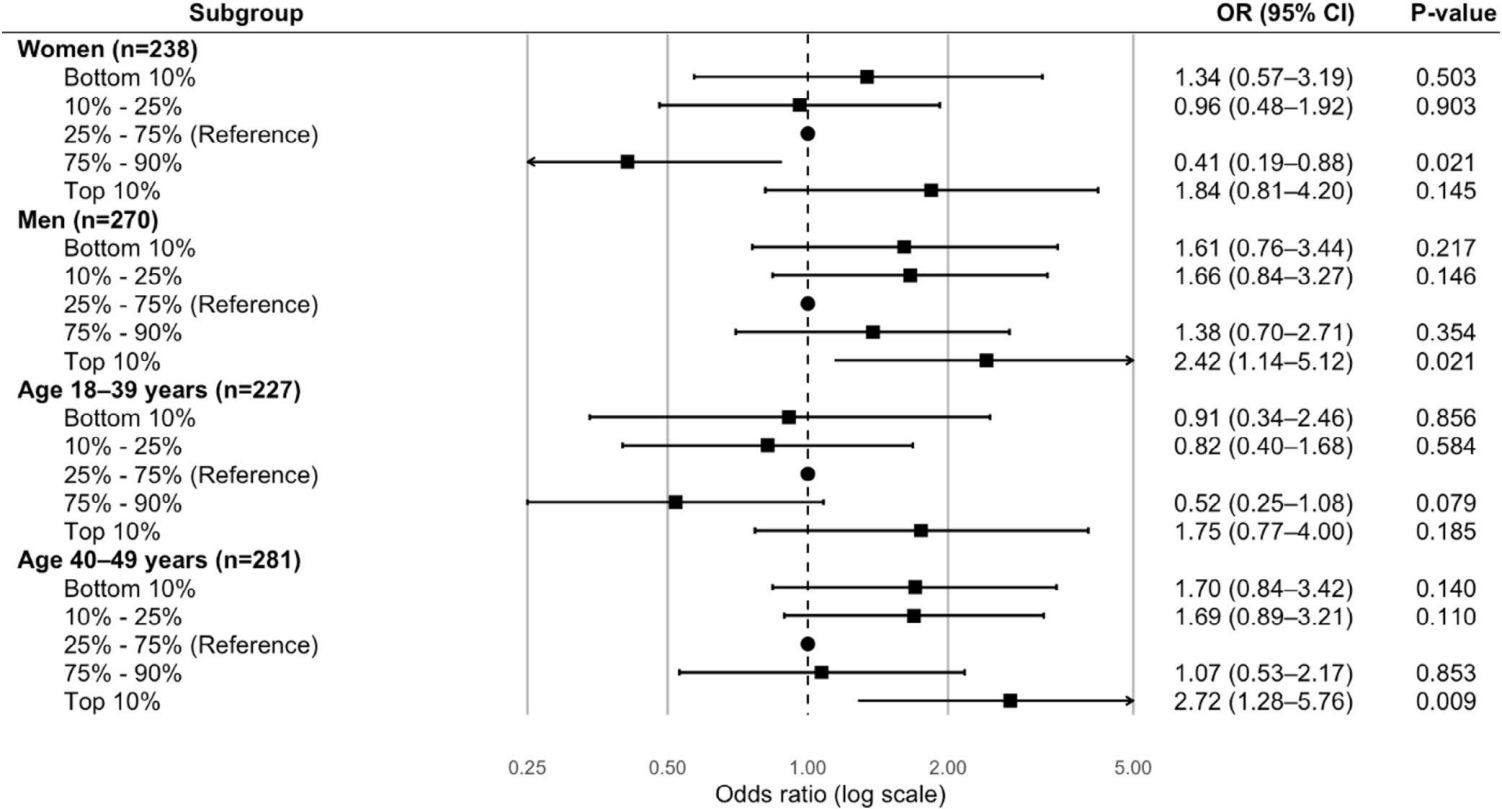
Association between physical activity and cryptogenic ischemic stroke, stratified by sex and age group. OR, odds ratio; CI, confidence interval. Model 3, fully adjusted for demographics, traditional and non‐traditional vascular risk factors.

In sex‐stratified analyses, women in the 75%–90% percentile of METs had a lower risk of CIS compared to the reference group; however, this association was observed only in the fully adjusted model. In contrast, among men, a significant association was identified for those in the top 10% of METs, likewise limited to the fully adjusted model when compared with male controls (Table S7, Figure 4).

### 3.4 Association Between PA and CIS According to Age Group

Among participants aged 18–39 years, no significant associations were detected across any adjustment models. In those aged 40–49 years, PA was associated with CIS after demographic adjustment for both the bottom 10% and top 10% of METs. After full adjustment, the association remained significant only for the top 10% of METs compared with controls (Table S8, Figure 4).

### 3.5 Association Between Sedentary Behavior and CIS

**FIGURE 5:**
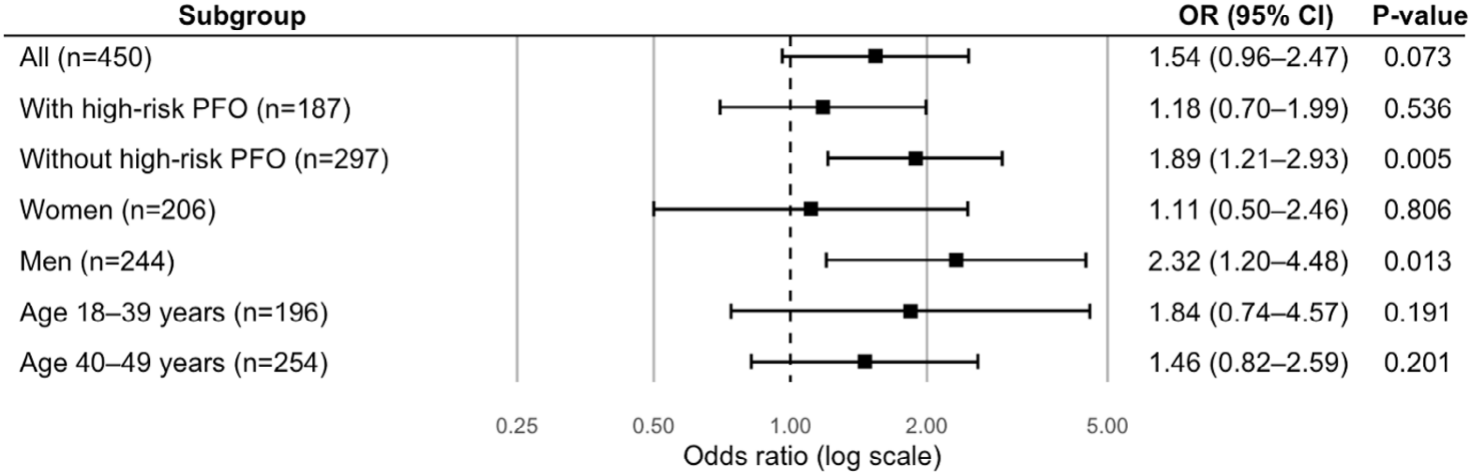
Association between sedentary behavior and cryptogenic ischemic stroke, stratified by patent foramen ovale status (PFO), sex, and age group. OR, odds ratio; CI, confidence interval. Model 3, fully adjusted for demographics, traditional and non‐traditional vascular risk factors.

In the overall cohort, sedentary behavior was significantly associated with CIS after adjustment for demographic and traditional risk factors, but not after including non‐traditional risk factors (Table S9, Figure 5).

No significant associations were found between sedentary behavior and CIS among patients with a high‐risk PFO compared to all controls under any adjustment model. In contrast, for patients without a high‐risk PFO, sedentary behavior showed a significant association with CIS in the fully adjusted model when compared to all controls (Table S9, Figure 5).

### 3.6 Association Between Sedentary Behavior and CIS According to Sex

In sex‐specific analyses, no significant associations between sedentary behavior and CIS were observed among female patients under any adjustment model when compared to female controls. Conversely, among male patients, sedentary behavior remained significantly associated with CIS across all models when compared to male controls (Table S10, Figure 5).

### 3.7 Association Between Sedentary Behavior and CIS According to Age Group

Among patients aged 18–39 years, sedentary behavior was significantly associated with CIS after adjustment for demographic and traditional risk factors, but not after including non‐traditional risk factors when compared to age‐matched controls. In contrast, no significant associations were observed in the 40–49 year age group under any adjustment model (Table S10, Figure 5).

### 3.8 Sensitivity Analyses

When restricting the analysis to case–control pairs with strictly population‐based controls, PA sensitivity analyses using population‐based controls supported the main findings (See supplementary results and Table S11).

## 4 Discussion

In this multicenter case–control study, we detected a robust independent association between PA and early‐onset CIS. While the impact of PA on stroke risk in general populations is well‐documented, our study provides novel evidence focusing specifically on cryptogenic etiology in young adults, a population where data has been previously lacking. This association remained significant in individuals with extreme PA and without a high‐risk PFO, in men, and in patients aged 40–49 years, even after adjusting for both traditional and non‐traditional vascular risk factors. For very low levels of PA, a significant association was observed only in individuals without a high‐risk PFO. By isolating CIS, our findings distinguish themselves from prior research that often combined all stroke etiologies, potentially masking mechanisms unique to the cryptogenic subgroup. Regarding sedentary behavior, significant associations were detected for individuals without a high‐risk PFO and for men.

Previous research has consistently shown that low PA increases stroke risk, with protective effects attributed to improved blood pressure, lipid regulation, and reduced inflammation [24, 25, 26]. In contrast to these findings, our results highlight that extreme levels of PA were most prominently associated with early‐onset CIS, while very low activity showed only limited associations. This divergence suggests that the relationship between PA and early‐onset CIS may not follow the same pattern as that observed in the general IS population. It is important to note, however, that previous studies primarily examined IS of any etiology, rather than CIS specifically.

Our percentile‐based approach to PA allows for a balanced comparison within this young population, although it does not directly measure adherence to absolute clinical thresholds. Current guidelines [27] recommend at least 150 min of moderate‐intensity or 75 min of vigorous‐intensity PA per week. While young adults generally exhibit higher adherence to these targets, our internal categorization highlights the risks associated with the extremes of the activity spectrum. We utilized the 25th–75th percentile range as a robust reference group to represent those achieving at least moderate activity levels. Although this wide range may conservatively dilute some associations, it ensured statistically stable comparisons and a clear contrast to the bottom and top 10% of PA.

Mechanistically, chronic inactivity creates a pro‐thrombotic environment via endothelial dysfunction and systemic inflammation [24]. Paradoxically, extreme exertion may trigger events; while habitual exercise is protective, transient risk rises during strenuous activity [28]. In young adults with PFO, vigorous exercise can facilitate paradoxical embolism via increased intrathoracic pressure during straining maneuvers [16]. While we focused on chronic exposure rather than acute triggers, we observed significant associations in those without high‐risk PFO, suggesting that PFO may contribute to the pathogenesis of CIS even when not meeting established high‐risk criteria. Beyond paradoxical embolism, chronic extreme endurance training has been linked to structural and electrical remodeling of the heart, and a higher incidence of atrial fibrillation (AF) [28]. Even when paroxysmal and asymptomatic, AF is recognized as a major risk factor for cardioembolic stroke [28], although the risk is generally regarded as low in younger patients without concomitant vascular risk factors. In our CIS cohort, AF was not present at study inclusion, although intermittent paroxysmal AF cannot be fully excluded, emphasizing that alternative mechanisms warrant further consideration. This aligns with the concept of a U‐shaped relationship between exercise and stroke risk, whereby moderate activity is uniformly protective, but very high activity levels may introduce specific cardiovascular vulnerabilities, a pattern also reflected in our cohort. High‐intensity exercise is associated with coronary calcification [29], though its relevance to the cerebrovascular system and CIS remains unclear and whether atherosclerotic mechanisms could partly explain the higher risk of CIS observed in the group with the highest PA in our study. Extreme PA can also cause arterial dissections via mechanical strain [30]. As dissections are a recognized cause of IS in young adults, they remain an important consideration when interpreting stroke mechanisms in very active individuals. Since dissections were excluded in our study, alternative mechanisms for CIS in active individuals warrant further exploration.

Beyond PA, sedentary behavior is an independent stroke risk factor, showing a dose–response relationship once daily thresholds are exceeded [22]. Importantly, this relationship appears particularly relevant for younger adults. For example, a large Canadian cohort study found that individuals under 60 years of age who spent 8 or more hours per day in leisure sedentary activity and in addition had low PA levels exhibited a 4.5‐fold higher long‐term risk of stroke compared to those who sat less than 4 h per day and/or maintained higher activity levels [13]. However, the study examined stroke of any kind, whereas our analysis specifically focuses on CIS, providing a more refined view of mechanisms in younger patients. Prolonged sitting induces hemodynamic and metabolic changes that favor a prothrombotic environment. Inactivity reduces lower‐limb blood flow and shear stress, leading to venous pooling, increased viscosity, and endothelial dysfunction. Since shear stress is a key regulator of vascular health, its reduction impairs antithrombotic and vasodilatory functions. Consistently, meta‐analytic evidence shows that prolonged uninterrupted sitting markedly reduces flow‐mediated dilation, underscoring how sedentary behavior promotes a pro‐inflammatory, hypercoagulable state [31]. In sedentary individuals with a PFO, venous thrombi formed during immobility may paradoxically cross into the arterial circulation, offering a plausible mechanism for CIS [32]. However, our findings did not provide evidence supporting this hypothesis.

A major strength of the SECRETO study is its strict adherence to a pre‐specified and published study protocol, combined with comprehensive and timely diagnostic assessments for all participants. To ensure a homogeneous study population and exclude stroke mimics, only patients with imaging‐confirmed CIS were included. Standardized examinations were performed across study sites, and detailed data were collected using validated, structured questionnaires, resulting in high granularity and minimal missing data, as both patients and controls were personally interviewed. The robustness of the results was further supported by sensitivity analyses, including restricting controls to population‐based individuals. The ability to account for multiple conventional and non‐traditional vascular risk factors in multivariable models adds to the validity of the findings. Finally, with participants recruited from 19 centers across Europe, the results can be considered generalizable to populations of European origin.

Nonetheless, some limitations need to be acknowledged. Although the study aimed to recruit all consecutive patients, some degree of selection bias toward those with milder strokes is possible. Yet, earlier studies have shown that younger IS patients typically present with lower NIHSS scores compared with older age groups [33]. As with any case–control study, our findings demonstrate associations but do not prove causality. Another limitation is that pre‐stroke PA was assessed retrospectively after the index stroke, which raises the possibility of recall bias. Selection bias may also arise from the fact that both patients and controls agreeing to participate in an extensive research protocol could differ systematically from non‐participants, for instance in terms of lifestyle habits or health awareness, potentially influencing the observed associations. To address this, we conducted sensitivity analyses restricted to population‐based controls, with results primarily in accordance with the main analyses.

## Author Contributions

**Shakar Kutal:** data curation, formal analysis, funding acquisition, methodology, software, project administration, writing – original draft, visualization. **Nilufer Yesilot:** conceptualization, writing – review and editing. **Tomi Sarkanen:** conceptualization, writing – review and editing. **Petra Redfors:** conceptualization, writing – review and editing. **Annika Nordanstig:** conceptualization, writing – review and editing. **Pauli Ylikotila:** conceptualization, writing – review and editing. **Marialuisa Zedde:** conceptualization, writing – review and editing. **Ulla Junttola:** conceptualization, writing – review and editing. **Lauri Tulkki:** conceptualization, writing – review and editing. **Kristina Ryliskiene:** conceptualization, writing – review and editing. **Radim Licenik:** conceptualization, writing – review and editing. **Phillip Ferdinand:** conceptualization, writing – review and editing. **Dalius Jatužis:** conceptualization, writing – review and editing. **Liisa Kõrv:** conceptualization, writing – review and editing. **Janika Kõrv:** conceptualization, writing – review and editing. **Alessandro Pezzini:** conceptualization, writing – review and editing. **Juha Sinisalo:** conceptualization, writing – review and editing. **Mika Lehto:** conceptualization, writing – review and editing. **Eva Gerdts:** conceptualization, writing – review and editing. **Jaana Autere:** conceptualization, writing – review and editing. **Ana Catarina Fonseca:** conceptualization, writing – review and editing. **André Paula:** conceptualization, writing – review and editing. **Ulrike Waje‐Andreassen:** conceptualization, writing – review and editing. **Bettina von Sarnowski:** conceptualization, writing – review and editing. **Tiina Sairanen:** conceptualization, writing – review and editing. **Turgut Tatlisumak:** conceptualization, writing – review and editing. **Juha Huhtakangas:** conceptualization, writing – review and editing. **Pekka Jäkälä:** conceptualization, writing – review and editing. **Jukka Putaala:** conceptualization, data curation, investigation, methodology, supervision, resources, validation, writing – review and editing. **Nicolas Martinez‐Majander:** conceptualization, data curation, investigation, supervision, resources, validation, writing – review and editing.

## Funding

Helsinki and Uusimaa Hospital District research fund (TYH2014407, TYH2018318); Academy of Finland (286,246, 318,075, 322,656); The Finnish Medical Foundation; The Sigrid Jusélius Foundation, Sahlgrenska University Hospital (ALFGBG‐726821); Emil Aaltonen Foundation. The funders had no role in study design, data collection, data analysis, data interpretation, writing of the report, or the decision to submit the article for publication.

## Conflicts of Interest

The authors declare no conflicts of interest.

## Supporting information


**Table S1:** Definitions and assessments methods for comorbidities.
**Table S2:** Comparison of baseline characteristics between patients in different physical activity categories.
**Table S3:** Comparison of baseline characteristics between female and male patients, and patients aged 18–39 years and 40–49 years.
**Table S4:** Metabolic equivalent categories of young cryptogenic ischemic stroke cases and stroke‐free control subjects included in the study, stratified by sex.
**Table S5:** Metabolic equivalent categories of young cryptogenic ischemic stroke cases and stroke‐free control subjects included in the study, stratified by age group.
**Table S6:** Odds ratios and 95% confidence intervals on the association between physical activity and cryptogenic ischemic stroke, stratified by patent foramen ovale status.
**Table S7:** Odds ratios and 95% confidence intervals on the association between physical activity and cryptogenic ischemic stroke, stratified by sex.
**Table S8:** Odds ratios and 95% confidence intervals on the association between physical activity and cryptogenic ischemic stroke, stratified by age group.
**Table S9:** Odds ratios and 95% confidence intervals on the association between sedentary behavior and cryptogenic ischemic stroke, stratified by patent foramen ovale status.
**Table S10:** Odds ratios and 95% confidence intervals on the association between sedentary behavior and cryptogenic ischemic stroke, stratified by sex and age group.
**Table S11:** Odds ratios and 95% confidence intervals on the association between physical activity, sedentary behavior, and cryptogenic ischemic stroke, based on case–control pairs in which controls were selected as population‐based only.

## Data Availability

The data that support the findings of this study are available from the corresponding author upon reasonable request.
