# Supplementary material for: Association Between Physical Activity and Cryptogenic Ischemic Stroke in Young Adults: A Case–Control Study
Source: Eur J Neurol. 2026 May 28;33(5):e70650. doi: 10.1111/ene.70650 (PMC13238723; doi:10.1111/ene.70650)
Supplement: Supplementary file 1 — Table S1: Definitions and assessments methods for comorbidities. Table S2: Comparison of baseline characteristics between patients in different physical activity categories. Table S3: Comparison of baseline characteristics between female and male patients, and patients aged 18–39 years and 40–49 years. Table S4: Metabolic equivalent categories of young cryptogenic ischemic stroke cases and stroke‐free control subjects included in the study, stratified by sex. Table S5: Metabolic equivalent categories of young cryptogenic ischemic stroke cases and stroke‐free control subjects included in the study, stratified by age group. Table S6: Odds ratios and 95% confidence intervals on the association between physical activity and cryptogenic ischemic stroke, stratified by patent foramen ovale status. Table S7: Odds ratios and 95% confidence intervals on the association between physical activity and cryptogenic ischemic stroke, stratified by sex. Table S8: Odds ratios and 95% confidence intervals on the association between physical activity and cryptogenic ischemic stroke, stratified by age group. Table S9: Odds ratios and 95% confidence intervals on the association between sedentary behavior and cryptogenic ischemic stroke, stratified by patent foramen ovale status. Table S10: Odds ratios and 95% confidence intervals on the association between sedentary behavior and cryptogenic ischemic stroke, stratified by sex and age group. Table S11: Odds ratios and 95% confidence intervals on the association between physical activity, sedentary behavior, and cryptogenic ischemic stroke, based on case–control pairs in which controls were selected as population‐based only. [file ENE-33-e70650-s001.docx]

**Supplementary appendix**

**Supplementary methods**

**Table s1:** Definitions and assessments methods for comorbidities

| **Variable** | **Definition** |
| --- | --- |
| Abdominal obesity | Waist-to-hip ratio >0.85 in women and >0.90 in men. |
| Current illicit drug use | Any illicit drug use within the past 12 months. Structured questionnaire.^1^ |
| Current smoking | Smoking at least one cigarette per day on average. |
| Depression | Feeling depressed at 2 weeks in the prior 12 months. Structured questionnaire.^2^ |
| Diabetes mellitus | Prior diagnosis of any diabetes and/or prior antidiabetic medication. |
| Heavy alcohol consumption | >7 (women) and >14 (men) units per week or at least an average of two times per month ≥5 (women) and ≥7 (men) units per instance (binge drinking). Adaptation of the WHO Alcohol, Smoking and Substance Involvement Screening Test.^1,3^ |
| History of cardiovascular disease | History of coronary heart disease, congestive heart failure, peripheral arterial disease, or atrial fibrillation. |
| History of chronic multisystem disorder | History of any of inflammatory bowel disease, autoimmune disease, chronic kidney/liver disease, or hematologic disease/thrombophilia. Patient history and medical records. |
| History of venous thrombosis | Patient history and medical records. |
| Hypercholesterolemia | Prior diagnosis of hypercholesterolemia or antilipemic medication. |
| Hypertension | Prior diagnosis of hypertension, prior antihypertensive medication or a mean of two office blood pressure measures 140/90 or over at study visit. |
| Low level of education | Either primary or lower secondary education, or upper secondary education |
| Malignancy | History of any malignancy based on comorbidities and VTE risk factors. Patient history and medical records. |
| Migraine with aura | Based on a validated migraine screener.^4^ |
| Obstructive sleep apnea syndrome | Patient history and medical records. |
| Psychological stress | At least several periods of stress at work or home (financial stress not considered). Structured questionnaire.^5^ |
| Unhealthy diet | Higher scores represent healthier diet, with a median cutoff of 24 points. A modified version of the Mediterranean Diet Score.^6^ |

**Statistical analysis**

For variables with more than 10 missing observations, specifically waist circumference (8.4%), hip circumference (8.7%), and diet score (14.9%), data were imputed using multivariable imputation by chained equations implemented in R. No imputation was required for control variables, as none had more than 10 missing values. The frequency of missing data was documented for all variables.

Baseline characteristics of cases and controls were compared using statistical methods appropriate for matched case-control designs. McNemar’s test was used for dichotomous variables, the paired t-test for normally distributed continuous or discrete variables, and the Wilcoxon signed-rank test for non-normally distributed continuous variables. Results are presented as absolute numbers (with percentages) or medians (with interquartile ranges).

Associations between physical activity categories and cryptogenic ischemic stroke were examined using conditional logistic regression, suitable for the matched case-control design. Three models were specified:

Model 1: adjusted for age, sex, and education level;

Model 2: adjusted for age, sex, education level, and traditional vascular risk factors;

Model 3: fully adjusted for age, sex, education level, traditional vascular risk factors, and non-traditional vascular risk factors.

Traditional vascular risk factors included hypertension, current smoking, abdominal obesity, heavy alcohol use, depression, unhealthy diet, and stress. Non-traditional vascular risk factors included migraine with aura, chronic multi-organ disorder, and illicit drug use.^7^ Diabetes, hypercholesterolemia, cardiovascular disease, obstructive sleep apnea, venous thromboembolism, and malignancy were not included in the regression models due to their low prevalence in the study population.

Additional conditional logistic regression analyses with similar adjustment models were performed stratified by sex, predefined age groups (18–39 and 40–49 years), and high-risk patent foramen ovale status. To assess robustness, sensitivity analyses compared cases with controls drawn exclusively from population-based registries, excluding hospital staff and unrelated proxy controls.

All statistical analyses were performed using International Business Machines Statistical Package for the Social Sciences Statistics for Windows (version 29.0.2; IBM Corp., Armonk, NY, USA) and RStudio (version 2024.04.2+764). A two-tailed p value <0.05 was considered statistically significant.

**Supplementary results**

**Table s2.** Comparison of baseline characteristics between patients in different physical activity categories.

| **Characteristic**  **(no. of patients with missing data if applicable)** |  |  | **Metabolic Equivalent Categories** | | |  |  |
| --- | --- | --- | --- | --- | --- | --- | --- |
|  | **25% - 75% (Reference, n=210)** | **Bottom 10%**  **(n=81)** | | **10% - 25%**  **(n=80)** | **75% - 90%**  **(n=77)** | | **Top 10 %**  **(n=85)** |
| **Age** | 40 (33–46) | 44 (38–46) | | 41 (36–46) | 41 (31–46) | | 40 (32–45) |
| **Low level of education (2)** | 91 [43.3] | 48 [60.0]* | | 37 [46.8] | 54 [70.1]* | | 64 [75.3]* |
| **Hypertension** | 67 [31.9] | 39 [48.1]* | | 25 [31.3] | 21 [27.3] | | 32 [37.6] |
| **Diabetes mellitus** | 6 [2.9] | 4 [4.9] | | 2 [2.5] | 0 [0.0] | | 4 [4.7] |
| **Cardiovascular disease**† | 4 [1.9] | 5 [6.2] | | 2 [2.5] | 1 [1.3] | | 3 [3.5] |
| **Current tobacco smoking (2)** | 45 [21.5] | 38 [47.5]* | | 23 [28.8] | 26 [33.8]* | | 41 [48.2]* |
| **Hypercholesterolemia** | 6 [2.9] | 5 [6.2] | | 1 [1.3] | 0 [0.0] | | 0 [0.0] |
| **Obesity**‡ | 107 [51.0] | 58 [71.6]* | | 49 [61.3] | 49 [63.6] | | 52 [61.2] |
| **Heavy alcohol use** | 26 [12.4] | 10 [12.3] | | 11 [13.8] | 10 [13.0] | | 13 [15.3] |
| **Depression (1)** | 67 [31.9] | 27 [33.3] | | 23 [28.8] | 21 [27.3] | | 25 [29.4] |
| **Unhealthy diet (2)** | 94 [45.0] | 48 [60.0]* | | 45 [56.3] | 39 [50.6] | | 46 [54.1] |
| **Obstructive sleep apnea (4)** | 3 [1.4] | 3 [3.8] | | 2 [2.5] | 0 [0.0] | | 5 [6.0]* |
| **Work or home related stress** | 105 [50.0] | 38 [46.9] | | 36 [45.0] | 42 [54.5] | | 44 [51.8] |
| **Migraine with aura** | 93 [44.3] | 36 [44.4] | | 32 [40.0] | 30 [39.0] | | 29 [34.1] |
| **Chronic multiorgan disorder§ (1)** | 30 [14.3] | 11 [13.6] | | 18 [22.5]* | 6 [7.8] | | 11 [13.1] |
| **Venous thromboembolism (2)** | 8 [3.8] | 3 [3.7] | | 1 [1.3] | 5 [6.5] | | 1 [1.2] |
| **Malignancy** | 4 [1.9] | 1 [1.2] | | 1 [1.3] | 3 [3.9] | | 1 [1.2] |
| **Illicit drug use** | 13 [6.2] | 3 [3.7] | | 4 [5.0] | 12 [15.6]* | | 9 [10.6] |

Data are n [%] or median (interquartile range).

*P-value <0.05 compared to the reference group

†Cardiovascular disease includes any of the following: coronary heart disease, chronic heart failure, peripheral artery disease, history of myocardial infarction, arterial thrombosis, aneurysm, or aortic or valvular diseases.

‡Waist-to-hip ratio >0.85 in women, >0.90 in men.

§Chronic multiorgan disorder includes any of inflammatory bowel disease (IBD), autoimmune disease (excluding IBD), chronic kidney or liver disease, or hematologic disease or thrombophilia

**Table s3.** Comparison of baseline characteristics between female and male patients, and patients aged 18-39 years and 40-49 years.

| **Characteristic**  **(no. of patients with missing data if applicable)** | **Women**  **(n=252)** | **Men**  **(n=281)** | **P-value** | **Age 18-39 years**  **(n=239)** | **Age 40-49 years**  **(n=294)** | **P-value** |
| --- | --- | --- | --- | --- | --- | --- |
| **Age** | 40 (31–45) | 42 (36–46) | 0.443 | 33 (27–37) | 45 (43–48) | 0.262 |
| **Low level of education (2)** | 131 [52.2] | 163 [58.2] | 0.163 | 131 [54.8] | 163 [55.8] | 0.816 |
| **Hypertension** | 80 [31.7] | 104 [37.0] | 0.202 | 56 [23.4] | 128 [43.5] | <0.001 |
| **Diabetes mellitus** | 6 [2.4] | 10 [3.6] | 0.426 | 3 [1.3] | 13 [4.4] | 0.033 |
| **Cardiovascular disease**† | 5 [2.0] | 10 [3.6] | 0.272 | 8 [3.3] | 7 [2.4] | 0.502 |
| **Current tobacco smoking (2)** | 68 [27.1] | 105 [37.5] | 0.011 | 82 [34.5] | 91 [31.1] | 0.406 |
| **Hypercholesterolemia** | 2 [0.8] | 10 [3.6] | 0.032 | 1 [0.4] | 11 [3.7] | 0.010 |
| **Obesity**‡ | 108 [42.9] | 207 [73.7] | <0.001 | 113 [47.3] | 202 [68.7] | <0.001 |
| **Heavy alcohol use** | 29 [11.5] | 41 [14.6] | 0.293 | 36 [15.1] | 34 [11.6] | 0.234 |
| **Depression (1)** | 84 [33.3] | 79 [28.1] | 0.192 | 79 [33.1] | 84 [28.6] | 0.264 |
| **Unhealthy diet (2)** | 117 [46.6] | 155 [55.4] | 0.044 | 124 [51.9] | 148 [50.7] | 0.784 |
| **Obstructive sleep apnea (4)** | 1 [0.4] | 12 [4.3] | 0.004 | 1 [0.4] | 12 [4.1] | 0.006 |
| **Work or home related stress** | 132 [52.4] | 133 [47.3] | 0.244 | 119 [49.8] | 146 [49.7] | 0.976 |
| **Migraine with aura** | 127 [50.4] | 93 [33.1] | <0.001 | 99 [41.4] | 121 [41.2] | 0.951 |
| **Chronic multiorgan disorder§ (1)** | 47 [18.7] | 29 [10.3] | 0.006 | 39 [16.4] | 37 [12.6] | 0.213 |
| **Venous thromboembolism (2)** | 10 [4.0] | 8 [2.9] | 0.474 | 7 [2.9] | 11 [3.8] | 0.607 |
| **Malignancy** | 4 [1.6] | 6 [2.1] | 0.642 | 4 [1.7] | 6 [2.0] | 0.756 |
| **Illicit drug use** | 16 [6.3] | 25 [8.9] | 0.270 | 25 [10.5] | 16 [5.4] | 0.031 |

Data are n [%] or median (interquartile range).

†Cardiovascular disease includes any of the following: coronary heart disease, chronic heart failure, peripheral artery disease, history of myocardial infarction, arterial thrombosis, aneurysm, or aortic or valvular diseases.

‡Waist-to-hip ratio >0.85 in women, >0.90 in men.

§Chronic multiorgan disorder includes any of inflammatory bowel disease (IBD), autoimmune disease (excluding IBD), chronic kidney or liver disease, or hematologic disease or thrombophilia

**Table s4.** Metabolic equivalent categories of young cryptogenic ischemic stroke cases and stroke-free control subjects included in the study, stratified by sex.

| **Metabolic Equivalent Category** | **Women**  **(252 pairs)** | | | **Men**  **(281 pairs)** | | |
| --- | --- | --- | --- | --- | --- | --- |
|  | **Cases n (%)** | **Controls n (%)** | **P-value** | **Cases n (%)** | **Controls n (%)** | **P-value** |
| **Bottom 10%** | 42 (16.7) | 26 (10.3) | 0.036 | 39 (13.9) | 28 (10.0) | 0.169 |
| **10% - 25%** | 42 (16.7) | 40 (15.9) | 0.899 | 38 (13.5) | 40 (14.2) | 0.905 |
| **25% - 75%** | 105 (41.6) | 123 (48.8) | 0.120 | 105 (37.4) | 144 (51.2) | 0.001 |
| **75% - 90%** | 28 (11.1) | 38 (15.1) | 0.229 | 49 (17.4) | 42 (14.9) | 0.477 |
| **Top 10%** | 35 (13.9) | 25 (9.9) | 0.184 | 50 (17.8) | 27 (9.6) | 0.007 |

**Table s5.** Metabolic equivalent categories of young cryptogenic ischemic stroke cases and stroke-free control subjects included in the study, stratified by age group.

| **Metabolic Equivalent Category** | **Age 18-39 years**  **(239 pairs)** | | | **Age 40-49 years**  **(294 pairs)** | | |
| --- | --- | --- | --- | --- | --- | --- |
|  | **Cases n (%)** | **Controls n (%)** | **P-value** | **Cases n (%)** | **Controls n (%)** | **P-value** |
| **Bottom 10%** | 26 (10.9) | 18 (7.5) | 0.256 | 55 (18.7) | 36 (12.2) | 0.027 |
| **10% - 25%** | 34 (14.2) | 35 (14.6) | 1.000 | 46 (15.6) | 45 (15.3) | 1.000 |
| **25% - 75%** | 102 (42.7) | 119 (49.8) | 0.142 | 108 (36.7) | 148 (50.3) | <0.001 |
| **75% - 90%** | 36 (15.1) | 40 (16.7) | 0.708 | 41 (13.9) | 40 (13.6) | 1.000 |
| **Top 10%** | 41 (17.2) | 27 (11.3) | 0.076 | 44 (15.0) | 25 (8.5) | 0.018 |

**Table s6.** Odds ratios and 95% confidence intervals on the association between physical activity and cryptogenic ischemic stroke, stratified by patent foramen ovale status.

|  | **Model 1: Adjusted for age, sex, and level of education.** | | **Model 2: Adjusted for age, sex, level of education, and traditional vascular risk factors§** | | **Model 3: Adjusted for age, sex, level of education, traditional and non-traditional vascular risk factors**¶ | |
| --- | --- | --- | --- | --- | --- | --- |
|  | **OR (95% CI)** | **P-value** | **OR (95% CI)** | **P-value** | **OR (95% CI)** | **P-value** |
| **All (531 pairs)** † |  |  |  |  |  |  |
| **Metabolic Equivalent percentiles** |  |  |  |  |  |  |
| **Bottom 10%** | 1.64 (1.05-2.57) | 0.030 | 1.34 (0.80-2.23) | 0.268 | 1.39 (0.80-2.39) | 0.241 |
| **10%-25%** | 1.21 (0.82-1.79) | 0.348 | 1.22 (0.80-1.87) | 0.361 | 1.26 (0.79-2.02) | 0.327 |
| **25%-75%** | Reference |  | Reference |  | Reference |  |
| **75%-90%** | 0.96 (0.64-1.42) | 0.821 | 0.87 (0.57-1.35) | 0.540 | 0.75 (0.46-1.21) | 0.234 |
| **Top 10%** | 1.61 (1.04-2.49) | 0.031 | 1.79 (1.11-2.89) | 0.018 | 2.07 (1.22-3.51) | 0.007 |
|  |  |  |  |  |  |  |
| **With high-risk PFO (195 cases)** ‡ |  |  |  |  |  |  |
| **Metabolic Equivalent percentiles** |  |  |  |  |  |  |
| **Bottom 10%** | 1.22 (0.70-2.14) | 0.480 | 1.00 (0.56-1.81) | 0.992 | 1.16 (0.62-2.19) | 0.645 |
| **10%-25%** | 1.15 (0.71-1.87) | 0.574 | 1.16 (0.71-1.91) | 0.554 | 1.20 (0.70-2.06) | 0.499 |
| **25%-75%** | Reference |  | Reference |  | Reference |  |
| **75%-90%** | 0.87 (0.52-1.47) | 0.607 | 0.87 (0.51-1.48) | 0.602 | 0.69 (0.39-1.23) | 0.209 |
| **Top 10%** | 1.71 (1.03-2.85) | 0.039 | 1.92 (1.14-3.25) | 0.015 | 1.72 (0.96-3.08) | 0.069 |
|  |  |  |  |  |  |  |
| **Without high-risk PFO (319 cases)** ‡ |  |  |  |  |  |  |
| **Metabolic Equivalent percentiles** |  |  |  |  |  |  |
| **Bottom 10%** | 2.11 (1.34-3.32) | 0.001 | 1.78 (1.09-2.91) | 0.020 | 1.76 (1.06-2.92) | 0.028 |
| **10%-25%** | 1.34 (0.87-2.08) | 0.184 | 1.35 (0.84-2.15) | 0.212 | 1.41 (0.87-2.28) | 0.164 |
| **25%-75%** | Reference |  | Reference |  | Reference |  |
| **75%-90%** | 1.26 (0.82-1.93) | 0.284 | 1.23 (0.79-1.93) | 0.366 | 1.10 (0.69-1.75) | 0.686 |
| **Top 10%** | 1.63 (1.03-2.59) | 0.037 | 1.63 (1.00-2.68) | 0.052 | 1.78 (1.07-2.94) | 0.026 |

OR, odds ratio; CI, confidence interval; PFO, patent foramen ovale.

†Analyzed using a conditional logistic regression, matching each case to a single control participant.

‡Analyzed using a binary logistic regression, comparing the selected cases to all controls.

§Traditional vascular risk factors include hypertension, current smoking, obesity, heavy alcohol use, depression, unhealthy diet, and stress.

¶Non-traditional vascular risk factors include migraine with aura, chronic multiorgan disease, and illicit drug use.

**Table s7.** Odds ratios and 95% confidence intervals on the association between physical activity and cryptogenic ischemic stroke, stratified by sex.

|  | **Model 1: Adjusted for age and level of education.** | | **Model 2: Adjusted for age, level of education, and traditional vascular risk factors**† | | **Model 3: Adjusted for age, level of education, traditional and non-traditional vascular risk factors**‡ | |
| --- | --- | --- | --- | --- | --- | --- |
|  | **OR (95% CI)** | **P-value** | **OR (95% CI)** | **P-value** | **OR (95% CI)** | **P-value** |
| **Women (238 pairs)** |  |  |  |  |  |  |
| **Metabolic Equivalent percentiles** |  |  |  |  |  |  |
| **Bottom 10%** | 1.79 (0.93-3.45) | 0.083 | 1.50 (0.69-3.23) | 0.307 | 1.34 (0.57-3.19) | 0.503 |
| **10%-25%** | 1.12 (0.63-1.97) | 0.702 | 1.08 (0.69-1.97) | 0.814 | 0.96 (0.48-1.92) | 0.903 |
| **25%-75%** | Reference |  | Reference |  | Reference |  |
| **75%-90%** | 0.66 (0.36-1.20) | 0.173 | 0.62 (0.32-1.18) | 0.146 | 0.41 (0.19-0.88) | 0.021 |
| **Top 10%** | 1.55 (0.80-3.02) | 0.193 | 1.84 (0.88-3.84) | 0.104 | 1.84 (0.81-4.20) | 0.145 |
|  |  |  |  |  |  |  |
| **Men (270 pairs)** |  |  |  |  |  |  |
| **Metabolic Equivalent percentiles** |  |  |  |  |  |  |
| **Bottom 10%** | 1.55 (0.84-2.89) | 0.165 | 1.24 (0.61-2.54) | 0.556 | 1.61 (0.76-3.44) | 0.217 |
| **10%-25%** | 1.28 (0.74-2.21) | 0.380 | 1.47 (0.78-2.74) | 0.232 | 1.66 (0.84-2.71) | 0.146 |
| **25%-75%** | Reference |  | Reference |  | Reference |  |
| **75%-90%** | 1.29 (0.75-2.21) | 0.353 | 1.30 (0.70-2.40) | 0.406 | 1.38 (0.70-2.71) | 0.354 |
| **Top 10%** | 1.71 (0.96-3.06) | 0.070 | 1.85 (0.96-3.56) | 0.068 | 2.42 (1.14-5.12) | 0.021 |

OR, odds ratio; CI, confidence interval.

†Traditional vascular risk factors include hypertension, current smoking, obesity, heavy alcohol use, depression, unhealthy diet, and stress.

‡Non-traditional vascular risk factors include migraine with aura, chronic multiorgan disease, and illicit drug use.

**Table s8.** Odds ratios and 95% confidence intervals on the association between physical activity and cryptogenic ischemic stroke, stratified by age group.

|  | **Model 1: Adjusted for age, sex, and level of education.** | | **Model 2: Adjusted for age, sex, level of education, and traditional vascular risk factors**† | | **Model 3: Adjusted for age, sex, level of education, traditional and non-traditional vascular risk factors**‡ | |
| --- | --- | --- | --- | --- | --- | --- |
|  | **OR (95% CI)** | **P-value** | **OR (95% CI)** | **P-value** | **OR (95% CI)** | **P-value** |
| **Age 18-39 years (227 pairs)** |  |  |  |  |  |  |
| **Metabolic Equivalent percentiles** |  |  |  |  |  |  |
| **Bottom 10%** | 1.42 (0.64-3.14) | 0.386 | 0.98 (0.39-2.43) | 0.960 | 0.91 (0.34-2.46) | 0.856 |
| **10%-25%** | 1.09 (0.60-1.99) | 0.778 | 0.96 (0.50-1.83) | 0.894 | 0.82 (0.40-2.46) | 0.584 |
| **25%-75%** | Reference |  | Reference |  | Reference |  |
| **75%-90%** | 0.72 (0.40-1.31) | 0.282 | 0.61 (0.31-1.18) | 0.139 | 0.52 (0.25-1.08) | 0.079 |
| **Top 10%** | 1.24 (0.65-2.35) | 0.520 | 1.32 (0.63-2.78) | 0.458 | 1.75 (0.77-4.00) | 0.185 |
|  |  |  |  |  |  |  |
| **Age 40-49 years (281 pairs)** |  |  |  |  |  |  |
| **Metabolic Equivalent percentiles** |  |  |  |  |  |  |
| **Bottom 10%** | 1.93 (1.11-3.36) | 0.020 | 1.65 (0.87-3.15) | 0.128 | 1.70 (0.84-3.42) | 0.140 |
| **10%-25%** | 1.33 (0.79-2.27) | 0.286 | 1.48 (0.82-2.67) | 0.194 | 1.69 (0.89-3.21) | 0.110 |
| **25%-75%** | Reference |  | Reference |  | Reference |  |
| **75%-90%** | 1.13 (0.65-1.97) | 0.662 | 1.10 (0.59-2.04) | 0.761 | 1.07 (0.53-2.17) | 0.853 |
| **Top 10%** | 1.91 (1.05-3.48) | 0.035 | 2.33 (1.19-4.59) | 0.014 | 2.72 (1.28-5.76) | 0.009 |

OR, odds ratio; CI, confidence interval.

†Traditional vascular risk factors include hypertension, current smoking, obesity, heavy alcohol use, depression, unhealthy diet, and stress.

‡Non-traditional vascular risk factors include migraine with aura, chronic multiorgan disease, and illicit drug use.

**Table s9.** Odds ratios and 95% confidence intervals on the association between sedentary behavior and cryptogenic ischemic stroke, stratified by patent foramen ovale status.

|  | **Model 1: Adjusted for age, sex, and level of education.** | | **Model 2: Adjusted for age, sex, level of education, and traditional vascular risk factors§** | | **Model 3: Adjusted for age, sex, level of education, traditional and non-traditional vascular risk factors**¶ | |
| --- | --- | --- | --- | --- | --- | --- |
|  | **OR (95% CI)** | **P-value** | **OR (95% CI)** | **P-value** | **OR (95% CI)** | **P-value** |
| **All (450 pairs)** † |  |  |  |  |  |  |
| **≥ 10 hours of daily sitting time** | 1.66 (1.14-2.43) | 0.009 | 1.66 (1.09-2.55) | 0.020 | 1.54 (0.96-2.47) | 0.073 |
|  |  |  |  |  |  |  |
| **With high-risk PFO (187 cases)** ‡ |  |  |  |  |  |  |
| **≥ 10 hours of daily sitting time** | 1.46 (0.92-2.34) | 0.111 | 1.40 (0.86-2.27) | 0.174 | 1.18 (0.70-1.99) | 0.536 |
|  |  |  |  |  |  |  |
| **Without high-risk PFO (297 cases)** ‡ |  |  |  |  |  |  |
| **≥ 10 hours of daily sitting time** | 2.09 (1.40-3.13) | <0.001 | 1.86 (1.21-2.86) | 0.005 | 1.89 (1.21-2.93) | 0.005 |

OR, odds ratio; CI, confidence interval; PFO, patent foramen ovale.

†Analyzed using a conditional logistic regression, matching each case to a single control participant.

‡Analyzed using a binary logistic regression, comparing the selected cases to all controls.

§Traditional vascular risk factors include hypertension, current smoking, obesity, heavy alcohol use, depression, unhealthy diet, and stress.

¶Non-traditional vascular risk factors include migraine with aura, chronic multiorgan disease, and illicit drug use.

**Table s10.** Odds ratios and 95% confidence intervals on the association between sedentary behavior and cryptogenic ischemic stroke, stratified by sex and age group.

|  | **Model 1: Adjusted for age and level of education.** | | **Model 2: Adjusted for age, level of education, and traditional vascular risk factors**† | | **Model 3: Adjusted for age, level of education, traditional and non-traditional vascular risk factors**‡ | |
| --- | --- | --- | --- | --- | --- | --- |
|  | **OR (95% CI)** | **P-value** | **OR (95% CI)** | **P-value** | **OR (95% CI)** | **P-value** |
| **Women (206 pairs)** |  |  |  |  |  |  |
| **≥ 10 hours of daily sitting time** | 1.30 (0.70-2.44) | 0.408 | 1.23 (0.61-2.45) | 0.567 | 1.11 (0.50-2.46) | 0.806 |
|  |  |  |  |  |  |  |
| **Men (244 pairs)** |  |  |  |  |  |  |
| **≥ 10 hours of daily sitting time** | 1.92 (1.19-3.11) | 0.008 | 2.32 (1.29-4.15) | 0.005 | 2.31 (1.20-4.48) | 0.013 |
|  |  |  |  |  |  |  |
| **Age 18-39 years (196 pairs)** |  |  |  |  |  |  |
| **≥ 10 hours of daily sitting time** | 2.76 (1.38-5.55) | 0.004 | 2.34 (1.05-5.27) | 0.037 | 1.84 (0.74-4.57) | 0.191 |
|  |  |  |  |  |  |  |
| **Age 40-49 years (254 pairs)** |  |  |  |  |  |  |
| **≥ 10 hours of daily sitting time** | 1.37 (0.86-2.18) | 0.188 | 1.49 (0.89-2.52) | 0.133 | 1.46 (0.82-2.59) | 0.201 |

OR, odds ratio; CI, confidence interval.

†Traditional vascular risk factors include hypertension, current smoking, obesity, heavy alcohol use, depression, unhealthy diet, and stress.

‡Non-traditional vascular risk factors include migraine with aura, chronic multiorgan disease, and illicit drug use.

**Sensitivity analyses**

When restricting the analysis to case–control pairs with strictly population-based controls, PA remained associated with early-onset CIS in patients within the top 10% of METs after adjustment for age and level of education. However, this association was no longer evident after further adjustment (Table s11). On the other hand, sedentary behavior showed a significant association with early-onset CIS in models adjusted for age, sex, level of education and traditional risk factors, but the association did not reach significance once non-traditional risk factors were included (Table s11).

**Table s11.** Odds ratios and 95% confidence intervals on the association between physical activity, sedentary behavior, and cryptogenic ischemic stroke, based on case-control pairs in which controls were selected as population-based only.

|  | **Model 1: Adjusted for age, sex, and level of education.** | | **Model 2: Adjusted for age, sex, level of education, and traditional vascular risk factors**† | | **Model 3: Adjusted for age, sex, level of education, traditional and non-traditional vascular risk factors**‡ | |
| --- | --- | --- | --- | --- | --- | --- |
|  | **OR (95% CI)** | **P-value** | **OR (95% CI)** | **P-value** | **OR (95% CI)** | **P-value** |
| **Metabolic Equivalent percentiles (296 pairs)** |  |  |  |  |  |  |
| **Bottom 10%** | 1.91 (1.07-3.40) | 0.028 | 1.49 (0.78-2.84) | 0.230 | 1.65 (0.82-3.31) | 0.162 |
| **10%-25%** | 1.09 (0.65-1.81) | 0.742 | 1.01 (0.58-1.74) | 0.983 | 1.06 (0.59-1.90) | 0.841 |
| **25%-75%** | Reference |  | Reference |  | Reference |  |
| **75%-90%** | 1.15 (0.66-2.00) | 0.615 | 0.97 (0.53-1.76) | 0.909 | 0.99 (0.53-1.85) | 0.982 |
| **Top 10%** | 1.55 (0.87-2.77) | 0.135 | 1.52 (0.81-2.83) | 0.189 | 1.62 (0.82-3.17) | 0.162 |
|  |  |  |  |  |  |  |
| **≥ 10 hours of daily sitting time (258 pairs)** | 1.75 (1.07-2.85) | 0.026 | 1.81 (1.05-3.12) | 0.033 | 1.73 (0.96-3.14) | 0.069 |

OR, odds ratio; CI, confidence interval.

†Traditional vascular risk factors include hypertension, current smoking, obesity, heavy alcohol use, depression, unhealthy diet, and stress.

‡Non-traditional vascular risk factors include migraine with aura, chronic multiorgan disease, and illicit drug use.

**Supplementary References**

1. Group WAW. The Alcohol, Smoking and Substance Involvement Screening Test (ASSIST): development, reliability and feasibility. *Addiction*. 2002;97:1183-1194.

2. Patten SB, Brandon-Christie J, Devji J, Sedmak B. Performance of the composite international diagnostic interview short form for major depression in a community sample. *Chronic Dis Can*. 2000;21:68-72.

3. Martinez-Majander N, Kutal S, Ylikotila P, et al. Association between heavy alcohol consumption and cryptogenic ischaemic stroke in young adults: a case–control study. *J Neurol Neurosurg Psychiatry*. 2025;96:114-121.

4. Martinez‐Majander N, Artto V, Ylikotila P, et al. Association between Migraine and Cryptogenic Ischemic Stroke in Young Adults. *Ann Neurol*. 2021;89:242-253

5. Khan M, Wasay M, O’Donnell MJ, et al. Risk Factors for Stroke in the Young (18–45 Years): A Case-Control Analysis of INTERSTROKE Data from 32 Countries. *Neuroepidemiology*. 2023;57:275-283.

6. Panagiotakos DB, Pitsavos C, Arvaniti F, Stefanadis C. Adherence to the Mediterranean food pattern predicts the prevalence of hypertension, hypercholesterolemia, diabetes and obesity, among healthy adults; the accuracy of the MedDietScore. *Prev Med*. 2007;44:335-340.

7. Putaala J, Martinez-Majander N, Leppert M, et al. Burden of Modifiable Risk Factors in Young-Onset Cryptogenic Ischemic Stroke by High-Risk Patent Foramen Ovale. *Stroke*. 2025;56:1428-1440.
